# Conservation of tRNA and rRNA 5-methylcytosine in the kingdom Plantae

**DOI:** 10.1186/s12870-015-0580-8

**Published:** 2015-08-14

**Authors:** Alice Louise Burgess, Rakesh David, Iain Robert Searle

**Affiliations:** School of Biological Sciences, The University of Adelaide, Adelaide, South Australia 5005 Australia; School of Agriculture, Food and Wine, The Waite Research Institute, The University of Adelaide, Adelaide, South Australia 5005 Australia; The University of Adelaide and Shanghai Jiao Tong University Joint International Centre for Agriculture and Health, Adelaide, Australia

**Keywords:** RNA 5-methylcytosine, Non-coding RNA, Ribosomal RNA (rRNA), Transfer RNA (tRNA), *Arabidopsis thaliana*, TRDMT1, DNMT2, TRM4, NOP2, NSUN5

## Abstract

**Background:**

Post-transcriptional methylation of RNA cytosine residues to 5-methylcytosine (m^5^C) is an important modification that regulates RNA metabolism and occurs in both eukaryotes and prokaryotes. Yet, to date, no transcriptome-wide identification of m^5^C sites has been undertaken in plants. Plants provide a unique comparative system for investigating the origin and evolution of m^5^C as they contain three different genomes, the nucleus, mitochondria and chloroplast. Here we use bisulfite conversion of RNA combined with high-throughput IIlumina sequencing (RBS-seq) to identify single-nucleotide resolution of m^5^C sites in non-coding ribosomal RNAs and transfer RNAs of all three sub-cellular transcriptomes across six diverse species that included, the single-celled algae *Nannochloropsis oculata,* the macro algae *Caulerpa taxifolia* and multi-cellular higher plants *Arabidopsis thaliana*, *Brassica rapa*, *Triticum durum* and *Ginkgo biloba*.

**Results:**

Using the plant model *Arabidopsis thaliana*, we identified a total of 39 highly methylated m^5^C sites in predicted structural positions of nuclear tRNAs and 7 m^5^C sites in rRNAs from nuclear, chloroplast and mitochondrial transcriptomes. Both the nucleotide position and percent methylation of tRNAs and rRNAs m^5^C sites were conserved across all species analysed, from single celled algae *N. oculata* to multicellular plants*.* Interestingly the mitochondrial and chloroplast encoded tRNAs were devoid of m^5^C in *A. thaliana* and this is generally conserved across *Plantae*. This suggests independent evolution of organelle methylation in animals and plants, as animal mitochondrial tRNAs have m^5^C sites. Here we characterize 5 members of the RNA 5-methylcytosine family in *Arabidopsis* and extend the functional characterization of TRDMT1 and NOP2A/OLI2. We demonstrate that nuclear tRNA methylation requires two evolutionarily conserved methyltransferases, TRDMT1 and TRM4B. *trdmt1 trm4b* double mutants are hypersensitive to the antibiotic hygromycin B, demonstrating the function of tRNA methylation in regulating translation. Additionally we demonstrate that nuclear large subunit 25S rRNA methylation requires the conserved RNA methyltransferase NSUN5. Our results also suggest functional redundancy of at least two of the NOP2 paralogs in *Arabidopsis.*

**Conclusions:**

Our data demonstrates widespread occurrence and conservation of non-coding RNA methylation in the kingdom *Plantae,* suggesting important and highly conserved roles of this post-transcriptional modification.

**Electronic supplementary material:**

The online version of this article (doi:10.1186/s12870-015-0580-8) contains supplementary material, which is available to authorized users.

## Background

5-methylcytosine (m^5^C) is a modification that occurs both on DNA and RNA. In DNA, m^5^C has been extensively studied due to its ease of detection and functional roles of DNA methylation in eukaryotes have been demonstrated for transcriptional silencing of transposons and transgenes, genomic imprinting and X chromosome inactivation (reviewed in [1]). While DNA appears to be devoid of other modifications [1], RNA has over 100 different modifications that have been identified in different species across all three domains of life [2–4]. Transfer RNAs (tRNAs) are heavily decorated with modifications that have been shown to stabilize secondary structure, affect codon identification and tRNA aminoacylation [5–8]. Of these modifications, m^5^C sites in tRNAs are commonly identified in the variable region and anticodon loop. In response to oxidative stress, m^5^C has been demonstrated to be dynamically modulated in yeast [9, 10] and m^5^C plays an important role in regulating tRNA stability and translation in mice under controlled conditions [11]. Furthermore, m^5^C is required for tRNA stability under heat stress and oxidative stress conditions in fruit flies [12]. In ribosomal RNAs (rRNA), m^5^C sites are thought to play a role in translation, rRNA processing and structure [13–15].

In eukaryotes, transfer RNA m^5^C methylation is catalysed by two RNA methyltransferases (RMTases); the first class of RMTase is known as tRNA specific methytransferase 4 (TRM4) or NOP2/Sun domain protein 2 (NSUN2), in yeast and animals respectively [11, 16, 17]. *NSUN2* mutations in humans are linked to inherited intellectual disability and this is thought to be mediated by increased cleavage of tRNAs by the ribonuclease angiogenin [18–22]. In mice, *nsun2* mutants are smaller and have reduced male fertility and have revealed a role in stem cell self-renewal and differentiation [23, 24]. Using phylogenetic analysis, two putative TRM4/NSUN2 paralogs, *TRM4A* and *TRM4B,* were identified in the *Arabidopsis* genome [25, 26], however these genes have not been characterized in plants. The second class of eukaryotic RMTase; Transfer RNA aspartic acid methyltransferase 1 (TRDMT1), also known as DNA methyltransferase 2 (DNMT2), has been shown to methylate tRNAs in *Drosophila*, *Arabidopsis* and *Homo sapiens*. In plants, only one m^5^C site in tRNA^Asp(GTC)^ at position C38 has been shown to be methylated by TRDMT1 [27]. While *Drosophila*, and *Arabidopsis trdmt1* mutants appear wild type under standard laboratory conditions, zebrafish deficient in TRDMT1 have reduced body size and impaired differentiation of specific tissues [27, 28]. In nuclear encoded eukaryotic tRNAs, m^5^C methylation has been commonly reported at six cytosine positions; C34, C38, C48, C49, C50 and C72 [2, 3, 18, 29–31]. Methylation has also been discovered on mitochondrial encoded tRNAs in humans and cows on several tRNAs at positions C48, C49 and C72 [29, 32]. However, the methylation status of chloroplast encoded tRNAs and rRNAs has not been previously reported.

Like tRNAs, ribosomal RNAs are highly conserved and have important roles in translation. The ribosome consists of two subunits, the large subunit (LSU) and the small subunit (SSU). The LSU is composed of three rRNA species in eukaryotes, and generally two rRNA species in prokaryotes, while the SSU contains only one rRNA species in both prokaryotes and eukaryotes [33–35]. The rRNA sequences are conserved, although the names of rRNA species are often not. Whereas rRNA methylation has not been investigated in plants, the location and enzymatic requirements of a few m^5^C sites in select organisms has been determined. For example, the human nuclear LSU rRNAs (28S and 5S) are methylated. The 28S rRNA contains two sites at C3782 and C4447 while 5S rRNA is methylated at C92 [30, 31, 36]. The orthologous yeast LSU 25S rRNA contains two sites at C2278 and C2870 [13, 15] and *E. coli* LSU 23S rRNA at C1962 [37] and SSU 16S rRNA at C967 [38] and C1407 [39]. Hamster mitochondrial SSU 13S rRNA also contains one m^5^C site [40], similarly mouse mitochondrial SSU 12S rRNA is methylated at position C911 [41]. Two RMTases that have been identified to methylate ribosomal RNA in eukaryotes are NOP2 (nucleolar protein 2) and RCM1 (rRNA cytosine methyltransferase 1). NOP2 methylates position C2870 and RCM1 methylates position C2278 in the LSU 25S rRNA in *Saccharomyces* [13, 15]. Yeast NOP2 is required for correct rRNA biosynthesis and processing [14] and *nop2* mutants are lethal. In contrast, yeast *rcm1* mutants are viable, however they are hypersensitive to anisomycin and this is thought to be due to structural changes being induced by methylation of rRNA [15]. While there is only one copy of the RCM1 homolog, referred to here as NSUN5 in *Arabidopsis*, there are three paralogs of *NOP2* in the *Arabidopsis* genome, *OLI2 (NOP2A)*, *NOP2B* and *NOP2C* [26]. One of these, *NOP2A*/*OLI2* was identified in a forward genetic screen for genes involved in compensation of cell size [42]. The methylation activity or m^5^C sites mediated by the three *Arabidopsis* NOP2 paralogs and NSUN5 are unknown. Another RMTase, which is related to the bacterial Fmu rRNA MTase was recently identified in *Arabidopsis* [43]. *Arabidopsis rnmt* (RNA methyltransferase) mutants had reduced global RNA methylation, indicating that it may methylate highly abundant rRNA transcripts.

Unlike animals, plant cells contain three evolutionary distinct genomes; nuclear, mitochondrial and chloroplast, thus providing a unique model for investigating m^5^C catalysis and biological function. The mitochondria is a striking example of how a prokaryotic translational machinery has adapted to input from eukaryotic translational machinery as nuclear, eukaryotic tRNAs are required to be imported into the mitochondria, as the mitochondria no longer has a full complement of tRNAs [44, 45]. tRNA sequences present in plants are dynamic, as there are multiple copies of each tRNA isodecoder and these can be lost within a genome or transferred from the chloroplast and mitochondrial genomes to the nucleus [46]. This gives rise to incidents where a nuclear encoded tRNA has an organelle-like sequence. It is unknown whether these “transferred” tRNAs are expressed after integration into a new genome as a systematic analysis of tRNA expression in plants is yet to be undertaken [47–49].

In this study, we describe single nucleotide resolution of post-transcriptionally modified cytosine residues in plant rRNA and tRNAs by combining RNA bisulfite conversion with second generation Illumina sequencing (RBS-seq). We report the identification of novel modified cytosines in *A. thaliana* nuclear transcribed tRNAs and that these sites are dependent on RMTases TRDMT1 and the previously undescribed *Arabidopsis* TRM4B. Additionally, we show these modified sites in nuclear tRNAs are conserved through evolution from the single celled algae *Nannochloropsis oculata* to multicellular higher plants. Interestingly, no m^5^C sites were detected in *Arabidopsis* chloroplast or mitochondrial tRNAs, which is in contrast to animal mitochondrial tRNAs. The function of tRNA methylation in regulating translation is demonstrated, as *trdmt1 trm4b* double mutants are hypersensitive to the antibiotic hygromycin B. Furthermore, we identify novel modified cytosines in nuclear, mitochondrial and chloroplast rRNAs. In *Arabidopsis* nuclear LSU 25S rRNA, m^5^C at C2268 was dependent on NSUN5, but methylation at C2860 was not found to be dependent on any particular NOP2 ortholog in *Arabidopsis*. Furthermore, RMTases responsible for methylation of tRNAs were not required for rRNA methylation, and vice versa indicating functional specialization of the RMTase family. These data represent the first high-resolution description of tRNA and rRNA modifications in the *plantae* kingdom and creates a platform to begin understanding the function, significance and evolution of non-coding RNA methylation.

## Results

### Detection and enrichment of transcribed tRNAs in *Arabidopsis thaliana*

To identify transcribed tRNAs in *A. thaliana* we implemented a two-step approach. First, a tRNA isodecoder consensus list was constructed to facilitate expression analysis and second, a tRNA enrichment protocol combined with Illumina deep-sequencing was developed similar to those recently described [50]. The tRNA isodecoder consensus approach was undertaken as there are over 640 predicted tRNA genes in *A. thaliana,* originating from the nuclear, mitochondrial and chloroplast genomes often with multiple identical isodecoder sequences that makes assigning IIlumina sequences to individual transcribed tRNA loci challenging. Using this consensus approach, the predicted *A. thaliana* tRNAs were resolved into 100 reference consensus sequences (Additional file 1: Table S1).

**Fig. 1:**
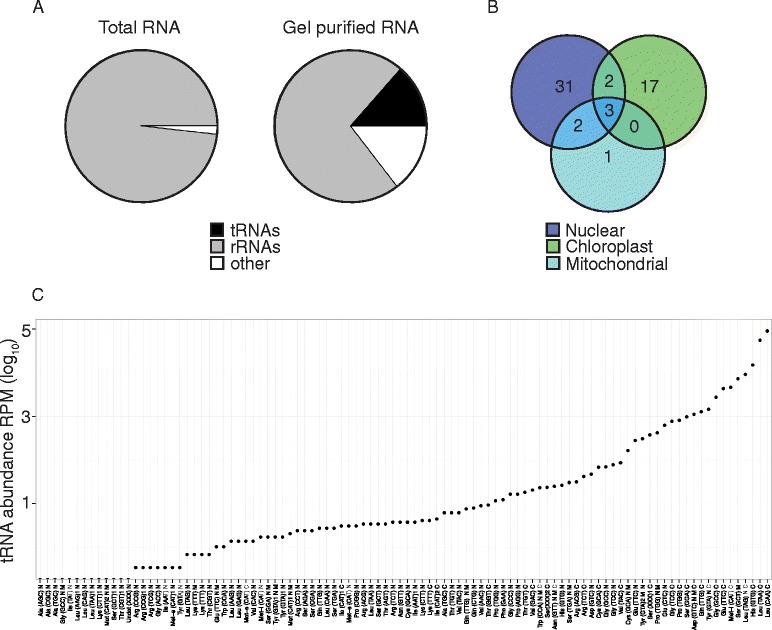
Efficient detection of *Arabidopsis* tRNAs by polyacrylamide gel purification and RNA-seq. **a** Comparison of Illumina sequencing reads from either total RNA or gel purified RNA shows an increase in reads mapping to tRNAs from 0.0007 to 13.58 %, respectively. Data from one representative biological replicate is shown. **b** Venn diagram showing detection of gel purified tRNA consensus sequences from nuclear, chloroplast and mitochondrial genomes. 56 out of 100 known tRNA consensus sequences were identified in our analysis. Overlapping circles indicate tRNAs that may originate from more than one genome (*n* = 3 biological replicates). **c** Consensus tRNAs display a wide range of expression levels with chloroplast (C) encoded sequences showing the highest expression levels compared to nuclear (N) and mitochondrial (M) sequences (1 replicate). Three of the tRNAs have undetermined anticodon sequences and are shown as (XXX). Minority isodecoders with diverged sequences from the majority isodecoder are designated by the number 1 or 2 after the anticodon. RBS-seq was used for (**a**) and (**b**) and RNA-seq was used in (**c**)

To identify transcribed tRNAs, we initially used total RNA to construct an Illumina library, deep-sequenced the library and aligned the sequenced reads to our tRNA consensus list. Only 0.0007 % of sequence reads aligned to tRNAs using this traditional approach. Therefore, we developed a method for tRNA enrichment prior to Illumina sequencing similar to those recently described (see Methods). Briefly, after separation of total RNA on a polyacrylamide gel, a region corresponding to the tRNAs was excised, RNA purified and then either bisulfite treated or directly used as template in library construction. Using this enrichment method, a nearly 20,000-fold increase in the sequence reads aligning to tRNAs was observed, when compared to using total RNA (Fig. 1a). Expression of 56 out of 100 isodecoder consensus sequences from all three genomes, nuclear, chloroplast and mitochondrial was observed using our RBS-seq data. Of these, seven tRNA sequences were ambiguously aligning with two or more genomes (Fig. 1b). A wide-range of tRNA transcript abundances were observed from our RNA-seq data, with chloroplast and mitochondrial derived tRNAs having the highest abundance (Fig. 1c). This is most likely a reflection of the high copy number of plastid and mitochondrial organelles per mesophyll cell.

### RBS-seq analysis to identify 5-methylcytosine (m^5^C) sites in tRNAs of *A. thaliana*

To identify m^5^C sites in tRNAs at single-nucleotide resolution, we performed bisulfite (BS) conversion on enriched tRNAs from wild type *Arabidopsis* that were combined with an *in vitro* transcribed Renilla Luciferase (R-Luc) mRNA BS conversion control lacking m^5^C. Complete BS conversion of R-Luc control results in no cytosines and serves as an important internal control. After BS conversion, Illumina libraries were constructed, deep-sequenced and aligned to *in silico* BS converted, cytosine to thymine, endogenous *Arabidopsis* tRNA consensus sequences and the R-Luc control. For a BS converted sample to pass our quality control standards, the R-Luc control required a minimum of 98 % conversion across the 178 cytosines present in the R-Luc mRNA BS conversion control (Additional file 1: Figure S1A). After passing R-Luc quality control, we then determined the global endogenous cytosine abundance. In all stranded RBS-seq libraries, global endogenous cytosine abundance was less than ~1 % compared to ~22 % for non-BS treated RNA-seq samples (Additional file 1: Figure S1B, S1C). Together these results demonstrated that bisulfite conversion of RNA cytosines was highly efficient using our method.

**Fig. 2:**
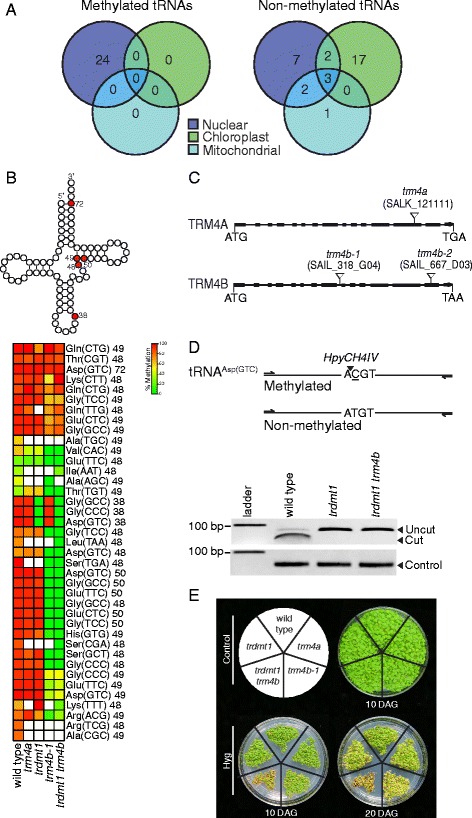
TRDMT1 and TRM4B methylate *Arabidopsis* nuclear encoded transfer RNAs. **a** Genomic origins of methylated and non-methylated tRNAs. Methylated tRNAs were only detected from the nuclear genome (3 biological replicates). **b** Above: clover-leaf representative secondary structure of tRNA indicating in red, the five cytosine positions methylated in wild type. Below: Heatmap showing percentage methylation of all cytosines detected in nuclear tRNAs of wild type, and mutants *trdmt1*, *trm4a*, *trm4b-1* and *trdmt1 trm4b* using RBS-seq. Cytosine positions are indicated next to tRNA isodecoders. White boxes represent cytosine positions with coverage less than five reads. (wild type 3 biological replicates, mutants *n* = 1). **c** Genomic structure of *trm4a* and *trm4b* mutants showing T-DNA insertions (triangles) in exons (filled boxes). **d** Analysis of RNA methylation by TRDMT1 at position C38 on BS treated tRNA^Asp(GTC)^ template. Above: Restriction maps of PCR amplified products showing the expected digest patterns of methylated and non-methylated template. Below: Cleavage of PCR amplified product by HpyCH4IV confirms C38 methylation in wild type as opposed to non-methylated C38 in *trdmt1* results in loss of HpyCH4IV restriction site. Loading control is undigested PCR product. **e** Hygromycin B stress assay. *Trdmt1 trm4b* double mutants and to a lesser extent, *trm4b-1* mutants display increased sensitivity to hygromycin B (Hyg) at 10 and 20 days after germination (DAG) compared to controls

To identify m^5^C sites in nuclear, chloroplast and mitochondrial *Arabidopsis* tRNAs, we aligned the Illumina RBS-seq reads against an *in silico* converted tRNA consensus list. *In silico* conversion involved converting all cytosines to thymines. 5-methylcytosine sites were then identified as cytosines that resist bisulfite conversion. These sites are to be noted as candidate m^5^C sites, as other types of modified cytosine can also be resistant to bisulfite conversion [29, 51]. We applied a threshold of at least 5 reads aligning to an individual tRNA consensus and a minimum of 20 % methylation. Using these parameters, we identified 24 methylated tRNAs and 32 non-methylated tRNAs out of a total of 56 (Fig. 2a, Additional file 1: Table S2). Interestingly, only nuclear encoded tRNAs were found to contain m^5^C sites, whereas non-methylated tRNAs were encoded by all three genomes.

Cytosine methylation of *Arabidopsis* nuclear tRNAs ranged from 23 to 100 %, and were consistent between the three biological replicates. 39 m^5^C sites were identified at 5 structural positions and are illustrated on the representative tRNA secondary structure at positions C38, C48, C49, C50 and C72 (Fig. 2b). Methylation at these sites is consistent with observations in other non-plant species [2, 3, 18, 29–31]. Next we examined the pattern of methylation in individual tRNA isodecoders. Seventeen tRNAs were identified with methylation at only 1 structural position, while the other remaining 7 tRNAs contained 2–5 m^5^C sites per tRNA. The most frequently methylated sites corresponded to structural positions C48, C49 and C50, indicating that methylation in this region may be important for tRNA structure or stability. tRNA^Asp(GTC)^ was the most highly methylated tRNA and was the only tRNA containing methylation at all 5 structural positions. The structure of tRNA^Asp(GTC)^ may require these additional m^5^C sites for greater stability or resistance to cleavage.

### Identification of TRM4B and TRDMT1 dependent m^5^C sites in nuclear tRNAs

To confirm the m^5^C sites in *Arabidopsis* nuclear tRNAs and determine the RMTases required for methylation, we identified mutants for the predicted *Arabidopsis* homologs of RMTases TRM4 and TRDMT1 and then performed RBS-seq on libraries enriched for tRNAs.

Two TRM4 paralogs were identified in the *Arabidopsis* genome [25] and we refer to them as TRM4A and TRM4B. T-DNA mutations in *TRM4A* or *TRM4B* were identified and the homozygous mutants characterized by semi-quantitative RT-PCR to demonstrate null expression (Fig. 2c and Additional file 1: Figure S2C) and show mutants are most likely complete loss of function. Mutants *trm4a*, and the two isolated T-DNA mutants for *TRM4B*; *trm4b-1 and trm4b-2* were grown on soil and appeared phenotypically similar to wild type like the previously characterized RMTase mutant *trdmt1* [27] (Additional file 1: Figure S2A). To test for divergent functions of TRM4A and TR4MB, the m^5^C single-nucleotide profile of tRNAs was determined in the mutants (Fig. 2b). In *trm4a*, the m^5^C profile was the same as wild type, showing that TRM4A is not required for methylation of any of the detected tRNAs. In contrast for *trm4b-1* and *trm4b-2,* a total of 18 sites had no detectable methylation and 7 sites had reduced methylation when compared to wild type (Fig. 2b and Additional file 1: Figure S3A). The sites that lost methylation or had reduced methylation corresponded to structural positions C48, C49 and C50 which is consistent with animal studies [2, 3, 18, 29–31].

Further investigation of the functional motifs of TRM4A and TRM4B by sequence alignment demonstrated that TRM4A is missing motif I (Additional file 1: Figure S4A). Motif I is essential for methyltransferase activity and is required for AdoMet binding and catalysis [52]. Loss of motif I in TRM4A most likely explains why no reduction in tRNA m^5^C levels was observed in *trm4a*. However we cannot exclude the possibility that TRM4A has other functional roles. As TRM4B contains all of the predicted motifs required for RMTase activity and there is a reduction in m^5^C tRNA methylation in the *trrm4b* mutants, this demonstrated that TRM4B is the functional homolog of TRM4/NSUN2 in *Arabidopsis thaliana*.

TRDMT1 was previously reported to methylate three tRNAs, tRNA^Asp(GTC)^, tRNA^Gly(GCC)^ and tRNA^Val(AAC)^ at structural position C38, in animals [11, 12, 27, 30] and tRNA^Asp(GTC)^ in *Arabidopsis* [27]. RBS-seq analysis of wild type *Arabidopsis* and *trdmt1* not only confirmed that TRDMT1 is required for position C38 methylation of tRNA^Asp(GTC)^ but is also required for C38 methylation of tRNA^Gly(CCC)^ and tRNA^Gly(GCC)^ in plants as these sites had no detectable methylation in *trdmt1*. In contrast to animals, position C38 of tRNA^Val(AAC)^ is not methylated in *Arabidopsis* (Additional file 1: Table S2). All other detected tRNAs were not methylated at position C38.

Nine m^5^C sites in nuclear tRNAs did not show a reduction of methylation in *trm4a-1, trm4b-1* or *trdmt1* single mutants when compared to wild type. These sites occur at structural positions C47, C48, C49 and C72 and are shown clustered together at the top of the heatmap (Fig. 2b). To exclude the possibility of functional redundancy of TRM4B and TRDMT1, we constructed a *trdmt1 trm4b* double mutant and then performed RBS-seq. All 9 sites were methylated in the double mutant and therefore we concluded that no functional redundancy of TRM4B and TRDMT1 for methylation of specific cytosine residues occurs in *Arabidopsis*. We cannot rule out the possibility that these 9 sites are cytosines with other RNA modifications that, like m^5^C, are also resistant to bisulfite conversion and therefore are independent of TRM4A, TRM4B, or TRDMT1 methylation.

To further demonstrate the reproducibility of our tRNA methylation data, we developed a rapid PCR-digestion assay to investigate individual m^5^C sites derived from BS treated RNA. Position C38 of tRNA^Asp(GTC)^ coincides with the restriction enzyme digestion site, ACGT, of HpyCH4IV*.* Methylation of C38 protects the site from BS conversion maintaining the HpyCH4IV site in methylated tRNA^Asp(GTC)^ derived PCR products*.* Therefore HpyCH4IV only cleaves tRNA^Asp(GTC)^ PCR products when position C38 is methylated. Methylation of tRNA^Asp(GTC)^ at position C38 by TRDMT1 was confirmed using the digestion assay on wild type and *trdmt1* BS treated RNA (Fig. 2d). As expected, C38 of tRNA^Asp(GTC)^ is not methylated in *trdmt1* or *trdmt1 trm4b* double mutants and is not cleaved by HpyCH4IV after BS treatment. The rapid digestion assay confirmed our RBS-seq data.

To test the role of tRNA m^5^C sites in regulating translation, the antibiotic hygromycin B, hereafter described as hygromycin, was used to perturb translation. Hygromycin alters the conformation of the A-site in the ribosome, which increases binding of tRNAs to the A-site, inhibits translocation and reduces translational fidelity [53]. The tRNA RMTases TRDMT1 and TRM4B mutants are expected to be more sensitive to hygromycin, as the loss of methylation is predicted to weaken the structural integrity of select tRNAs and increase the ability of hygromycin to bind and ‘lock’ tRNAs in the A-site, stopping translocation. Therefore we tested this expectation by growing wild type and mutants on control and hygromycin containing plates. Both *trm4b* and t*rdmt1 trm4b* double mutants displayed increased sensitivity to hygromycin at 10 and 20 days after germination (DAG) when compared to the controls (Fig. 2e). The sensitivity of *trm4b* mutants to hygromycin is more apparent at 20 days DAG than at 10 DAG. As a number of tRNAs lose methylation in *trm4b* and t*rdmt1 trm4b* mutants (Fig. 2b) and previous reports that loss of methylation affects tRNA structure, we attribute the hygromycin sensitivity of the mutants to a modified tRNA structure and the increased interaction between these tRNAs and the A-site of the ribosome reducing translation.

### Identification of m^5^C sites in *Arabidopsis* nuclear, chloroplast and mitochondrial ribosomal RNAs

To identify m^5^C sites in rRNAs from *A. thaliana*, we first constructed a list of rRNA sequences to represent all rRNAs from nuclear, mitochondrial and chloroplast genomes (Additional file 1: Table S3). Then we *in silico* bisulfite converted all cytosines to thymines before aligning the RBS-seq data. RBS-seq transcriptome libraries from total RNA were sequenced and efficient bisulfite conversion of cytosine residues was determined as previously described (Additional file 1: Figure S1A, S1B and Methods).

**Fig. 3:**
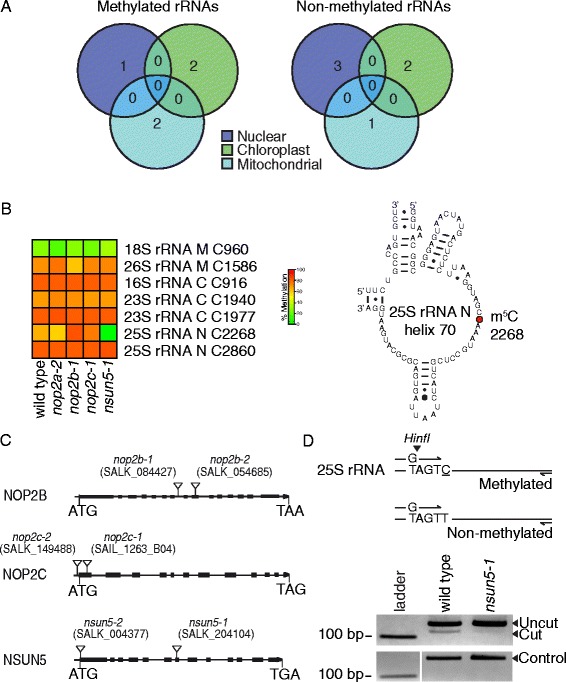
NSUN5 methylates C2268 in *Arabidopsis* nuclear LSU 25S rRNA. **a** Genomic origins of methylated and non-methylated rRNA species. Methylated rRNAs were detected from all three genomes (3 biological replicates). **b** Left: Heatmap showing percentage methylation of cytosines in nuclear (N), chloroplast (C) and mitochondrial (M) rRNA sequences in wild type and mutants *nop2a-2*, *nsun5-1*, *nop2b-1* and *nop2c-1*. Cytosine positions are indicated next to rRNAs (3 biological replicates). Right: Partial secondary structure of 25S nuclear LSU rRNA helix 70 (domain IV) showing the cytosine position 2268 in red, which is methylated by NSUN5. **c** Genomic structure of *nop2b*, *nop2c* and *nsun5* mutants showing T-DNA insertions (triangles) in exons (filled boxes). **d** Analysis of RNA methylation by NSUN5 at position C2268 on BS treated nuclear LSU 25S rRNA template. Above: Restriction maps of dCAPS amplified products showing the expected digest patterns of methylated and non-methylated template. The 25S_rRNA_F dCAPS primer contains a G mismatch at position four to generate a HinfI restriction site when C2268 is methylated. Below: Cleavage of PCR amplified product by HinfI confirms C2268 methylation in wild type as opposed to non-methylated C2268 in *nsun5-1* results in loss of HinfI restriction site. Loading control is undigested PCR product

We identified a total of 7 m^5^C sites in the nuclear LSU 25S rRNA, chloroplast SSU 16S, LSU 23S and mitochondrial SSU 18S and LSU 26S rRNAs (Fig. 3a, b). This pattern is in contrast to tRNA methylation, which was only detected on nuclear tRNAs (Fig. 2a). Each methylated rRNA contained one m^5^C site except for the nuclear LSU 25S and chloroplast LSU 23S rRNAs that each contained two m^5^C sites (Fig. 3b). Of the 7 m^5^C sites, 6 were highly methylated in all three biological replicates and the average wild type methylation levels ranged from 66 to 82 %. In contrast, one m^5^C site, C960 in mitochondrial SSU 18S rRNA, was lowly methylated, with an average of 28 % methylation (Fig. 3b). There were 6 rRNA species that were not methylated (Fig. 3a and Additional file 1: Table S3).

### NSUN5 is required for m^5^C at position C2268 in nuclear LSU 25S rRNA

Two positions, C2268 and C2860, in nuclear LSU 25S rRNA were highly methylated in our RBS-seq datasets and both sites occur in the conserved domain IV of the large rRNA subunit in helices 70 and 89, respectively. Recently, for the orthologous positions C2278 and C2870 in the yeast nuclear LSU 25S rRNA, the RMTases RCM1 and NOP2 were shown to be required for methylation, respectively [13, 15]. Therefore, we predicted that the *Arabidopsis* homolog of RCM1, described here as NSUN5, and NOP2 paralogs described here as NOP2A/OLI2, NOP2B and NOP2C would be required for m^5^C at these sites [25, 42]. To test these predictions we performed RBS-seq on *nsun5*, *nop2a*, *nop2b* and *nop2c* mutants (Fig. 3c, Additional file 1: Figure S2B, S2C).

To test if NSUN5 is required for m^5^C at position C2268 of nuclear LSU 25S rRNA we performed RBS-seq on total RNA from *nsun5-1* and wild type (Fig. 3b). Methylation was reduced from 66 % in wild type to 2 % in *nusn5-1* at position C2268 and methylation was not reduced at any other rRNA m^5^C sites. Similar results were obtained for a second, independent allele, *nsun5-2* (Additional file 1: Figure S3D). Methylation of C2268 was reduced to 29 % in *nsun5-2*. The low level of background methylation in *nsun5-2* may be due to low levels of NSUN5 expression in this mutant. While no transcripts were detected spanning the T-DNA insertion site, (Additional file 1: Figure S2C) spurious splicing may be occurring at low frequency to produce a small amount of functional, truncated protein. To confirm reduced methylation at position C2268 in nuclear 25S rRNA in *nsun5* mutants, we developed a restriction enzyme digestion of PCR products using a dCAPs (derived cleaved amplified polymorphic sequences) primer derived from BS treated 25S rRNA. Cytosine methylation of C2268 retains the HinfI restriction site and the enzyme cleaves the PCR products in wild type (Fig. 3d and Additional file 1: Figure S3E). A reduction of C2268 methylation in *nsun5-1* and *nusn5-2* was observed by reduced cleavage of PCR products. Together these results demonstrate that C2268 25S rRNA is methylated by NSUN5 in *Arabidopsis*.

Next we tested if NOP2A, NOP2B or NOP2C were required for methylation at C2860 of nuclear LSU 25S rRNA by RBS-seq from the mutants (Fig. 3b and Additional file 1: Figure S3D). All mutants, *nop2a*, *nop2b* and *nop2c* had wild type levels of methylation at C2860 25S rRNA, suggesting these RMTases do not methylate this site or are functionally redundant. To address this question, we attempted to identify *nop2a nop2b* double mutants, however these double mutants could not be identified from a segregating population. This suggests that NOP2A and NOP2B may act redundantly and are essential for plant viability. Sequence alignment of NOP2A, NOP2B and NOP2C revealed that NOP2B is missing motif IV, which is predicted to be involved in release of methylated RNA from the enzyme [54, 55] and NOP2C has an altered motif N1, which is involved in RNA binding, but is not essential for RMTase activity, as TRM4 homologs do not contain this motif [56] (Additional file 1: Figure S4B). Further research is required to uncover the RMTase(s) responsible for this m^5^C site and the redundancy of NOP2 paralogs in *Arabidopsis*. We also tested if the tRNA RMTases TRM4A, TRM4B and TRDMT1 methylate the remaining 6 m^5^C sites in rRNAs by RBS-seq from the mutants, *trm4a*, *trm4b-1, trdmt1, trdmt1 trm4b* and wild type (Additional file 1: Figure S3C). As expected, no reduction in rRNA methylation levels for the 7 m^5^C sites was observed in the mutants. Similarly, to demonstrate NOP2A and NSUN5 are rRNA specific and do not methylate tRNAs, we performed RBS-seq from both *nop2a-2* and *nsun5-2.* No reductions in m^5^C tRNA sites were observed (Additional file 1: Figure S3B).

### tRNA and rRNA m^5^C sites are conserved from single-celled algae to multicellular plants

To test if methylated sites in nuclear tRNAs and organelle rRNAs are conserved through evolution, we constructed tRNA enriched RBS-seq libraries from six organisms; the single-celled algae, *N. oculata,* the multicellular macro algae *C. taxifolia,* and four vascular plants, the monocotyledonous plant *T. durum, the* dicotyledonous plants *A. thaliana* and *B. rapa* and the evolutionarily distinct *ginkgophyte plant G. biloba*. First, to identify transcribed tRNAs in non-*Arabidopisis* species, we mapped RNA-seq and RBS-seq to both our *Arabidopsis* tRNA isodecoder consensus sequences (Additional file 1: Table S1) and unique tRNA sequences from the closest relative with annotated tRNAs from the PlantRNA Database [49]. Similarly to construct species-specific rRNA references, we performed RNA-seq from total RNA from the five organisms and aligned the reads to either *Arabidopsis* rRNA references, species-specific rRNA references, or an *Arabidopsis*-rRNA guided assembled reference (Additional file 1: Table S3). These species-specific rRNA references were then utilized to align and annotate subsequent RBS-seq reads.

**Fig. 4:**
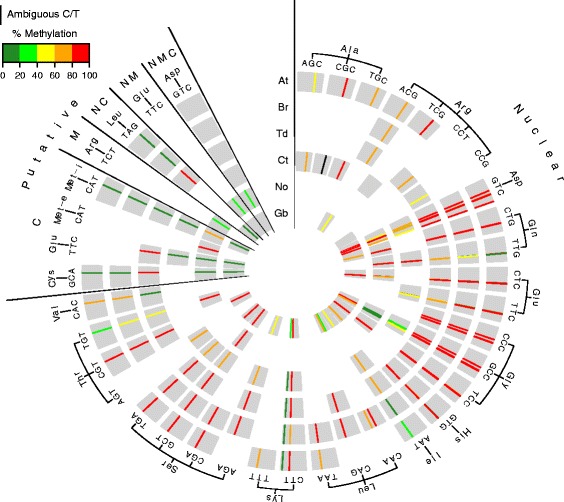
Conservation of tRNA methylation in Kingdom *Plantae.* **a** Concentric circles from outer to inner represent *Arabidopsis thaliana* (At), *Brassica rapa* (Br), *Triticum durum* (Td), *Nannochloropsis oculata* (No), *Caulerpa taxifolia* (Ct) and *Ginkgo biloba* (Gb) tRNAs, respectively. The circles are split into two major sections for nuclear encoded tRNAs and tRNAs with putative genomic origins (nuclear-N, chloroplast-C, mitochondrial-M). In each circle, individual tRNA consensus sequences are indicated as thick grey arcs and are organized alphabetically by amino acid, and then by anticodon. Specific tRNAs sequences for each species were aligned based on structural positions corresponding to the 72 bp representative tRNA structure. Cytosines that are methylated in at least one of the 6 species analysed are shown as a color-coded percentage methylation bar. The percentage methylation scheme used, Green = lowly methylated (0–40 %), red = highly methylated (80–100 %). Absence of a methylation bar indicates that the corresponding position in the tRNA does not contain a cytosine in that species. A black bar at position 49 in tRNA^Ala(CGC)^ in Ct represents an ambiguous nucleotide which may be a C or T at this position. tRNAs that were not detected in the RBS-seq are not shown (*Arabidopsis thaliana-* 3 biological replicates and all other plant species 1 replicate)

To test for conservation of m^5^C of tRNAs we performed RBS-seq on tRNA enriched libraries from *N. oculata, C. taxifolia*, *T. durum*, *B. rapa*, *A. thaliana*, and *G. biloba* and detected 35, 30, 51, 48, 56 and 34 tRNA isodecoders respectively (Fig. 4, Additional file 1: Table S2 and Table S4). Of these tRNAs, 30 were nuclear tRNAs, which are for the greater part methylated across all six species and the remaining 8 were putative chloroplast or mitochondrial tRNAs methylated in only one of the two species, *T. durum* or *N. oculata*. As these tRNAs were only methylated in one of the six species this may reflect chloroplast or mitochondrial tRNAs recently integrated into the nuclear genome of *T. durum* or *N. oculata*. Together these data demonstrate that methylation of chloroplast or mitochondrial encoded tRNAs is rare in the Kingdom *Plantae* and m^5^C methylation of tRNAs is generally restricted to nuclear-encoded tRNAs.

Detailed analysis of the 30 conserved nuclear tRNA isodecoders identified a total of 51 methylated positions. These 51 sites were divided into three classes, class one contained 35 highly conserved sites across all six species, class two contained 5 highly conserved sites in five species and the other species contained a single-nucleotide polymorphism (SNP) and the third class contained 11 sites which are methylated in at least one species and not methylated in the other species. Class two that contained SNPs, were either transitions (C > T) or transversions (C > G) at the methylated positions. An example of a transversion occurs in tRNA^Asp(GTC)^. At position C50 in tRNA^Asp(GTC)^ in *C. taxifolia* had a transversion from C to G, abolishing an otherwise highly conserved m^5^C site. The G transversion was confirmed by using RNA-seq. An example of class three, m^5^C site reduction in one species, was position C48 of tRNA^Glu(CTC)^. While *T.durum* and *G.biloba* had low levels of methylation (22–40 %) at C48, three other species were not methylated at this site, despite the presence of a cytosine residue in non-BS converted RNA.

Within class three, containing conserved cytosine residues methylated in at least one species, a noteworthy example was tRNA^Gln(TTG)^ which contained methylated positions in all species however sites were not conserved. For example, in *T. durum* and *N. oculata* positions C48 and C49 were both methylated however in the other tested species only C48 or C49 was methylated, but not both sites despite the presence of cytosines at these positions. This site variability was also identified by Blanco et al*.* [18], as mice are methylated at one site in tRNA^Gln(TTG)^, while humans are methylated at two sites. A clearer understanding of the other ribonucleotide modifications near these tRNA positions may provide further insight into these observations.

We also identified two additional m^5^C structural positions, C34 and C68 in tRNA^Leu(CAA)^ and tRNA^Lys(CTT)^ of *B. rapa* and *G. biloba,* respectively*,* that were not methylated in other species. tRNA^Leu(CAA)^ position C34 methylation was only detected in *B. rapa* and *G. biloba* at 89 and 20 %, respectively. The variation of methylation at this position may be due to environmental factors, as methylation at this site in yeast was previously shown to be altered under oxidative stress conditions [10]. It is predicted that tRNA^Leu(CAA)^ position C34 is methylated in *Arabidopsis* but we did not detect *Arabidopsis* tRNA^Leu(CAA)^ in our datasets. For tRNA^Lys(CTT)^ position C68, *G. biloba* had 25 % methylation while *A. thaliana*, *B. rapa* and *T. durum* had very low methylation (below our 20 % methylation threshold). Similarly, methylation at nearby structural positions C67 and C69 in other tRNAs has also been reported in humans [30].

**Fig. 5:**
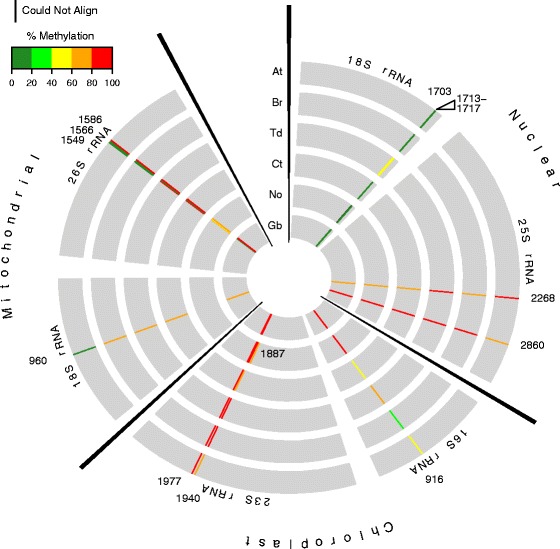
Conservation of rRNA methylation in Kingdom Plantae. **a** Concentric circles from outer to inner represent *Arabidopsis thaliana* (At), *Brassica rapa* (Br), *Triticum durum* (Td), *Nannochloropsis oculata* (No), *Caulerpa taxifolia* (Ct) and *Ginkgo biloba* (Gb) rRNAs, respectively. The circles are split into three sections for nuclear, chloroplast and mitochondrial encoded rRNAs. In each circle, individual rRNA sequences are represented as thick grey bars. The nucleotide positions for each rRNA species are based on alignment with the corresponding *Arabidopsis* consensus sequences. Cytosines that are methylated in at least one of the 6 species analysed are shown as a color-coded percentage methylation bar. In the percentage methylation scheme used, Green = lowly methylated (0–40 %), red = highly methylated (80–100 %). Absence of a methylation bar indicates that the corresponding position in the rRNA does not contain a cytosine in that species. Open triangle indicates where consecutive cytosines are methylated in *Triticum durum*. The black methylation bar at position 1703 in *Nannochloropsis oculata* SSU 18S rRNA shows where the sequence could not be aligned to the *Arabidopsis* reference sequence (At 3 replicates and other species 1 replicate)

Conservation of rRNAs m^5^C sites was tested amongst all six organisms, *N. oculata, C. taxifolia*, *T. durum*, *B. rapa*, *A. thaliana*, and *G. biloba,* by RBS-seq from total RNA. A total of 8 highly conserved m^5^C sites in nuclear, chloroplast and mitochondrial structural positions of LSU and SSU rRNAs were identified (Fig. 5 and Additional file 1: Table S3 and Table S5). Interestingly, methylation of LSU 25S rRNA cytosines C2268 and C2860, which are predicted to be dependent upon homologs of NSUN5 and NOP2A/NOP2B/NOP2C, respectively are conserved in all six species [13, 15]. Six of these 8 m^5^C sites were highly conserved in methylation percentage and position across all tested species except C916 in SSU 16S chloroplast rRNA for which the methylation across species ranged from 31 to 87 %. The remaining two highly conserved sites, mitochondrial C960 in SSU 18S rRNA and C1549 in LSU 26S rRNA were highly methylated in four of the six tested species*.* A further eight m^5^C sites, were species specific of which 6, C1703 and C1713-1717, occurred in a 15 bp region on *T. durum* nuclear SSU 18S rRNA and the other two methylated sites, C1566 mitochondrial SSU 18S rRNA and C1887 chloroplast LSU 23S rRNA occurred only in *N. oculata.* The five clustered m^5^C sites in 18S rRNA maybe attributed to BS non-conversion events as a result of strong secondary structure of the rRNA. The remaining species-specific sites in *N. oculata* may reflect species-specific factors regulating translation by ribosomes.

## Discussion

Here, we show that the post-transcriptional modification 5-methylcytosine is only detected on nuclear-encoded tRNAs of plants however methylation of rRNAs occurs in transcripts from all three organelles*.* Strong conservation of tRNA and rRNA methylated sites were observed in species ranging from single-celled algae to multicellular plants. Furthermore, in *Arabidopsis thaliana,* the evolutionarily conserved RNA methyltransferases TRM4B and TRDMT1 were found to be required for tRNA methylation at multiple nucleotide sites, while NSUN5 specifically methylates nuclear LSU 25S rRNA at position C2268.

Our study detected 39 candidate sites for 5-methylcytosine in *Arabidopsis* nuclear tRNAs and an additional 20 m^5^C sites were detected across diverse plant species and all sites except one are new discoveries in plants. The majority of m^5^C sites were found at positions within tRNA secondary structure known to have 5-methylcytosine in animals [2, 3, 18, 29–31], broadly supporting existing expectations of the role of m^5^C in modulating tRNA function [2]. An emerging facet of tRNA biology in both plants and animals is their processing into smaller regulatory RNAs [57–61], and TRDMT1- mediated addition of m^5^C has been demonstrated to protect tRNAs against heat and oxidative stress-mediated cleavage in *Drosophila* [12]. Likewise, methylation by TRM4/NSUN2 in humans and mouse has been demonstrated to protect tRNAs from oxidative stress induced cleavage [18]. Together, this data provides a wealth of m^5^C sites mediated by TRDMT1 and TRM4B that can now be interrogated for a role of this phenomenon in plants. Future experiments will determine if increased cleaved tRNA fragments are observed in the RMTase mutants and testing these mutants under various environmental conditions may highlight additional roles for these genes in modulating stress responses.

Detection of m^5^C sites on only nuclear tRNAs in *Arabidopsis* is consistent with the mitochondrial and chloroplast genomes being derived from alpha-proteobacteria [62] and cyanobacterial ancestors [63], respectively. Complementary to our data, m^5^C sites were not detected on tRNAs from bacterium *Escherichia coli* and *Bacillus subtilis* [3]. In contrast, six mitochondrial tRNAs in bovine [32] and five mitochondrial tRNAs in human [29, 64] contain methylation at positions C48, C49 and C72. These data suggest that methylation of mitochondrial tRNAs may have evolved independently in animals since the last common ancestor between plants and animals. A lack of methylation of mitochondrial and chloroplast tRNAs was generally conserved across diverse plant species. Three notable exceptions were chloroplast-like tRNA^Cys(GCA)^, tRNA^Glu(TTC)^ and tRNA^Leu(TAG)^ in *T. durum* that we detected methylation however no methylation was observed in the homologues of other plants species. One interpretation of these observations is that the three chloroplast-like tRNAs of *T. durum* represent recent DNA integration events into the nucleus. After nuclear integration and transcription, these tRNAs are methylated by RMTases, presumably TRM4B, TRDMT1 or RCMT9.

Many mitochondrial genes and tRNAs are often incorporated into nuclear genomes and accordingly lost from the organelle genomes over time [46]. As a result, to obtain the full complement of tRNAs required for translation of mitochondrial encoded proteins, requires the import of tRNAs from the nucleus. Nine tRNA isoacceptors are predicted to be imported from the nucleus to the mitochondrion in *Arabidopsis* [44] and several of these tRNAs, such as tRNA^Gly(CCC)^ were found to be methylated in our data. We speculate that m^5^C methylation by TRM4B and/or TRDMT1 of these mitochondrial-imported nuclear tRNAs occurs in the cytoplasm before import into the mitochondria. Methylation of nuclear, eukaryotic tRNAs that are imported into the mitochondria implies they are not inherently incompatible with the prokaryotic mitochondrial translation machinery.

Nine putative cytosine RMTase enzymes including TRDMT1/DNMT2 are encoded in the *Arabidopsis* genome of which we demonstrate TRM4B and TRDMT1 methylate tRNAs and not rRNAs [25, 26]. Duplication of TRM4 resulted in two paralogs, TRM4A and TRM4B in *Arabidopsis.* TRM4B retains methyltransferase activity on tRNAs while TRM4A contains a deletion of motif I which is required for target cytosine binding [52]. We cannot rule out that TRM4A contains other regulatory functions, for example regulating m^5^C stability or modulating accessibility of m^5^C sites to RNA binding proteins. TRM4B methylation of tRNAs at positions C48, C49 and C50 is consistent with the fact that animal and yeast homologues also methylate these tRNA structural positions [2, 3, 16, 18, 29–31]. NSUN2/TRM4 in human has been found to methylate tRNA(s) at position C72 [30]. In contrast, we identified only one C72 methylated position in tRNA^Asp(GTC)^, which was independent of TRM4B. TRDMT1 has been previously described as a tRNA C38 specific RMTase, in animals and methylates tRNA^Asp(GTC)^, tRNA^Gly(GCC)^ and tRNA^Val(AAC)^ [11, 12, 30]. We confirmed this C38 specific observation in *Arabidopsis* by not only detecting the previously described tRNA^Asp(GTC)^, but also two new tRNAs, tRNA^Gly(GCC)^ and tRNA^Gly(CCC)^. The importance of these methylated sites in tRNAs is illustrated in other organisms, as loss of TRM4 and TRDMT1 results in reduced abundance of mature tRNAs and translational efficiency in mice [11]. In addition, tRNA m^5^C sites mediated by TRM4 in yeast are required for tolerance to the antibiotic paromomycin, which is an aminoglycoside, like hygromycin B [65]. Similarly our data of *trm4b* mutants increased sensitivity to hygromycin B when compared to wild type suggests TRM4B methylated tRNAs have a role in translational efficiency. Interestingly the loss of both TRDMT1 and TRM4B resulted in a severe sensitivity to hygromycin B. There are only three tRNAs which we found to be methylated by both TRDMT1 and TRM4B suggesting that the m^5^C mediated structure of these three tRNAs is important for translation. Translation is tightly regulated in order for organisms to adapt quickly to environmental stresses, such as oxidative and heat stress [10, 66]. Alterations in translation in *Arabidopsis trdmt1 trm4b* mutants may affect translational regulation under stress conditions and reduce plant fitness.

Our identification of m^5^C sites at tRNA structural positions C48, C49, C50 and C72 independent of either TRM4B or TRDMT1 is similar to recent observations in mouse using RBS-seq and raises the possibility that another RMTase methylates these sites [18]. This is in contrast to humans where all tRNA m^5^C sites are dependent on either TRM4 or TRDMT1 [18]. We propose that the additional tRNA RMTase in plants is the closest TRM4 homologue RCMT9 (At5g66180). This hypothesis could be tested by identification of *rcmt9* mutants and performing RBS-seq on enriched tRNAs as described here. An alternative hypothesis is that the TRM4 and TRDMT1 independent m^5^C sites are not m^5^C sites but other cytosine modifications that are resistant to BS conversion [29, 51]. This could be tested by performing mass spectrometry on purified tRNAs from plant *trm4b trdmt1* double mutants and determining the presence or absence of m^5^C. In *nsun2 trdmt1* double mutant mice, RNA m^5^C levels were reduced by at least 90 % compared to wild type mice [11]. It is unclear whether this indicates that additional m^5^C sites in tRNAs remain, or if the detected m^5^C was derived from contaminating rRNA. We favour the first hypothesis that these TRM4B and TRDMT1 independent sites are *bona fide* m^5^C sites as they are in tRNA structural positions which normally contain m^5^C and that these sites are methylated by Arabidopsis RCMT9, which shares sequence homology with TRM4 homologues [25].

Our approach not only detected methylated sites in RNAs but also provides a quantitative measure of the percentage of transcripts having this modification. This allowed us to undertake a quantitative comparative analysis of methylation in more than 200 individual sites in tRNAs and 50 individual sites in rRNAs amongst diverse plants. Interestingly both the percent methylation and specific sites in tRNAs and rRNAs were broadly conserved across the six plant species. This strong conservation strongly supports the functional importance for these m^5^C sites in roles such as regulating the structure and stability of rRNAs and tRNAs [2].

Our study detected 7 candidate sites for 5-methylcytosine in *Arabidopsis* nuclear, chloroplast and mitochondrial LSU and SSU rRNAs, all of them novel in plants. Many of these high-confidence sites were found at positions within rRNA regions known to have 5-methylcytosine in animals and bacteria [2, 12, 14, 33–36]. Of note, we did not detect LSU 5S rRNA methylation in any plant species analyzed, while in contrast, this rRNA species is methylated by TRM4/NSUN2 in HeLa cells [30, 31]. It is intriguing that while plant chloroplast and mitochondrial tRNAs are devoid of m^5^C, organelle rRNAs contain m^5^C. It is unknown whether the rRNAs are methylated inside the chloroplast and mitochondria, or if they are exported to allow addition of modifications from nuclear derived modifying enzymes before they are imported back into the organelles. *Arabidopsis* RMTases NOP2B and RNMT/FMU are both predicted to localize in chloroplasts [26]. This suggests that these RMTases methylate the rRNA inside the organelles. Further study is required on the location of the RMTases, to confirm these findings and to assess where catalysis of m^5^C occurs.

There are five RMTases present in the *Arabidopsis* genome, which are predicted to methylate rRNA, based on sequence similarity to rRNA RMTases in other organisms. In this study, we investigated the rRNA m^5^C sites requiring the RCM1 homolog, NSUN5 and the NOP2 paralogs NOP2A, NOP2B and NOP2C. Here we showed that *Arabidopsis* NSUN5 is required for methylation of C2278 in nuclear LSU 25S rRNA. Interestingly, the yeast NOP2 ortholog in *Arabidopsis,* NOP2A was not found to be required for m^5^C at C2860 of nuclear LSU 25S rRNA. We hypothesise that this may be due to functional redundancy with the other NOP2 paralogs in the *Arabidopsis* genome, NOP2B and NOP2C. It is uncertain if the paralogs NOP2B and NOP2C are functional RMTases. NOP2B lacks motif IV, which is involved in release of target RNA after methylation by motif VI [54, 55]. In yeast, a point mutation in motif IV of the conserved residue Cys^424^ in NOP2 leads to accumulation of mutant *nop2* protein-RNA complexes and cell death [54, 67]. It is possible that NOP2B is utilizing a Cys residue contained in a highly diverged, non-conforming motif IV to evade cell death. An alternative possibility is that although the m^5^C site is conserved, that another RMTase in the genome is responsible for methylation at this site. The most promising candidate for this possibility is the *Arabidopsis* homolog of bacterial Fmu, RNMT, which is predicted to methylate rRNA [25, 43].

Conservation of the enzymes and methylation sites in rRNA across species suggests conservation of function. The possible functions include, regulation of protein synthesis, stability and maturation of rRNA and translational fidelity. It remains to be seen if the phenotype of *nop2a* can be linked to any specific m^5^C sites, or alterations in rRNA processing. The rRNA m^5^C sites and the mutants identified in this work provide a platform to launch studies into the roles of specific rRNA m^5^C sites under different environmental conditions.

## Conclusions

This comprehensive characterization of the tRNA and rRNA methylation profiles in plants uncovered nuclear specific methylation of tRNAs, while rRNAs are methylated from all three genomes. The method of enriching for tRNAs combined with RBS-seq on wild type and mutants allowed us to identify m^5^C sites dependent on NSUN2/TRM4 and to extend the known target range of TRDMT1 to an additional two tRNAs at position C38 in plants. We also determined that NSUN5 is required for m^5^C at C2278 in nuclear LSU 25S rRNA and uncovered functional redundancy among the *Arabidopsis* NOP2 paralogs, NOP2A, NOP2B and NOP2C, as loss of one of these three enzymes is insufficient to remove any rRNA m^5^C sites, while loss of both NOP2A and NOP2B appears lethal. *Arabidopsis* RMTase enzymes are encoded in the nuclear genome. This suggests movement of either the nuclear encoded RMTase enzymes to organelles, or transport and re-import of organelle rRNAs. We favour the former hypothesis as the *Arabidopsis* Fmu-like RNMT and NOP2B are predicted to be located in organelles [26], which suggests that they methylate rRNA inside the mitochondria and chloroplast, and that NOP2B acts redundantly with NOP2A and NOP2C. While several mitochondrial tRNAs are methylated in vertebrates [29, 32, 64], our data suggests that like bacterial tRNAs, plant mitochondrial and chloroplast tRNAs are not methylated [3]. This suggests that vertebrates gained the ability to methylate mitochondrial tRNAs during evolution. We discovered high levels of conservation of tRNA and rRNA methylation across diverse plant species, including the sites shown to be methylated by TRDMT1, TRM4B and NSUN5 in *Arabidopsis*, indicating that these enzymes are most likely responsible for methylation at these sites in other *Plantae*. The conservation of RMTases and m^5^C sites strongly suggests important, conserved functions, which deserve investigation. We investigated the function of tRNA m^5^C sites using the antibiotic hygromycin B. Loss of both TRDMT1 and TRM4B resulted in increased sensitivity of mutants to the antibiotic hygromycin B, suggesting that tRNA m^5^C sites affect tRNA structure in a combinatorial manner. Our data provides the foundation and characterization of *Arabidopsis* genetic mutants needed to further probe the functions of RNA m^5^C in plants.

## Methods

### Plant material and growth conditions

*A. thaliana* (Columbia ecotype) and *B.rapa* plants were grown in Phoenix Biosystem controlled environment rooms at 21°C under metal halide lights that provided a level of PAR (photosynthetic active radiation) of 110 μmol of photos/m^2^/s. Plants were grown under long day photoperiod conditions of 16 h light and 8 h darkness on soil (Debco Seedling raising mix) or ½ MS media supplemented with 1 % sucrose. *G. biloba* was grown at the Botanic Gardens of Adelaide (34.9181° S, 138.6107° E, Australia). *C. taxifolia* was grown in artificial seawater at 25°C under fluorescent lights that provided PAR of 80 μmol of photos/m^2^/s. *N. oculata* was grown in artificial seawater supplemented with nitrogen and phosphorous under fluorescent lights that provided PAR of 40 μmol of photos/m^2^/s to log phase. For *Arabidopsis* hygromycin B assays, seeds were sown on control and hygromycin B [15 ug/mL] supplemented ½ MS media with 1 % sucrose. Hygromycin B was purchased from A.G. Scientific, Inc.. Plants were photographed with a Canon PowerShot G15 camera at 10 and 20 days after germination (DAG).

Mutant alleles described are; *nop2a-2 (oli2-2)* (SALK_129648), *nop2b-1* (SALK_084427), *nop2b-2* (SALK_054685), nop2c-1 (SAIL_1263_B04), *nop2c-2* (SALK_149488), *nsun5-1* (SALK_204104), *nsun5-2* (SALK_004377), *trdmt1* (SALK_136635), *trm4a* (SALK_121111), *trm4b-1* (SAIL_318_G04) and *trm4b-2* (SAIL_667_D03). The *trdmt1 trm4b* double mutants were generated using the *trdmt1* and the *trm4b-1* mutant alleles. The *nop2a nop2b* double mutants were generated using the *nop2a-2 (oli2-2)* and the *nop2b-1* mutant alleles. Primers used to identify homozygous T-DNA mutants are provided (Additional file 1: Table S6).

Nucleotide sequence data for the following genes are available from The Arabidopsis Information Resource (TAIR) database under the following accession numbers: NOP2A/OLI2 (At5g55920), NOP2B (At4g26600), NOP2C (At1g06560), NSUN5 (At5g26180), TRDMT1 (At5g25480), TRM4A (At4g40000), TRM4B (At2g22400).

### RNA isolation and bisulfite conversion of RNA

Total RNA was isolated from either 10 day old *A. thaliana* seedlings, or *A. thaliana* floral buds, *T. durum* flag leaf, *B. rapa* and *G. biloba* shoot apexes, *C. taxifolia* fronds or log phase growth *N. oculata* cells using the Spectrum Plant total RNA kit (SIGMA-ALDRICH) and contaminating DNA removed using DNase (SIGMA-ALDRICH). To enrich for tRNAs, 10μg of total RNA was separated on a 10 % polyacrylamide gel, the region containing 65–95 nts was removed and RNA was purified. Either total RNA or purified tRNAs were used for library construction using Illumina’s TruSeq RNA sample kit v2, as per the manufacturer’s instructions. As bisulfite treated RNA is sheared, bisulfite treated samples were quickly processed after addition of the fragmentation buffer. For bisulfite conversion, 200 pg of control *in vitro* transcribed Renilla luciferase (R-Luc) RNA was added to either 2 μg of total RNA or 200 ng of purified tRNAs and converted with sodium metabisulfite (SIGMA-ALDRICH) as previously described [29, 51]. Bisulfite treated total RNA or purified tRNAs were used as template for Illumina library construction as described above. Illumina sequencing was performed on a MiSeq platform at ACRF, Adelaide. For a description of the sequenced libraries, see (Additional file 1: Table S7).

### Sequence read mapping and methylation analysis

Sequences were trimmed for adapters and filtered for low quality reads using CLC Genomics Workbench (Qiagen). In order to identify tRNAs and to reduce mapping ambiguity, we collapsed identical and highly similar tRNA isodecoder sequences from The Arabidopsis Information Resource [68] to create a reference consensus list of 100 tRNA isodecoders (Additional file 1: Table S1). For tRNAs from other species, both the Arabidopsis reference consensus list and unique tRNA sequences obtained from the closest relative available in the PlantRNA Database [49], were used. rRNA sequences were obtained from the NCBI Refseq database where available. For species without available rRNA sequences, RNA-seq reads were aligned to the *Arabidopsis* RNA reference sequence for the corresponding rRNA subunit and a consensus was derived in order to obtain species-specific SNPs (Additional file 1: Table S3). RBS-seq reads of tRNAs and rRNAs were mapped to *in silico* converted reference sequences, while RNA-seq reads were mapped to unconverted sequences. CLC Genomics Workbench (Qiagen) was used to align sequence reads to the corresponding tRNA and rRNA reference sequences. In order to compare structural positions in tRNAs with different sequences and lengths, the representative structure model was used [69]. For rRNAs, the reference sequences of all plant species analysed were aligned to the *Arabidopsis* references and the numbering was based on the nucleotide position of the corresponding *Arabidopsis* rRNA reference sequence.

In order to identify methylated cytosines, non-conversion of a cytosine in read sequences was taken to indicate the presence of m^5^C. Renilla Luciferase *in vitro* transcribed mRNA lacking m^5^C was used to ensure that conversion efficiency was greater than 98 %. To ensure robust detection of methylated sites in tRNAs and rRNAs, a minimum of 5 reads coverage was required and a minimum methylation level of 20 % (≤80 % conversion rate). Percentage methylation at specific positions was calculated as the number of mapped cytosines divided by the combined total number of mapped cytosines and mapped thymines. Heatmaps showing percentage methylation were created in R [70], using the R package “Pretty heatmaps” [71].

### Methyl-chop PCR methylation assay

cDNA derived from the bisulfite treated RNA was used to generate PCR products from tRNA Asp(GTC) and helix 70 of 25S nuclear rRNA using the primers forward Asp tRNA_At_FWD and reverse Asp tRNA_At_REV and primers forward 25S_rRNA_F and reverse 25S_rRNA_R, respectively. The 25S_rRNA_F dCAPS primer contains a G mismatch at position four from the 3′ end to generate a HinfI restriction site. PCR products were digested with HpyCH4IV or HinfI restriction enzymes (New England Biolabs), respectively. The tRNA^Asp(GTC)^ 72bp PCR product is digested by *Hpy*CH4IV resulting in two digestion products of 35bp and 37bp if C38 is methylated, and is undigested if C38 is non-methylated and thus converted to T38 by bisulfite treatment, causing the HpyCH4IV restriction enzyme site to be lost. The 25S rRNA PCR product is 155bp in length. When C2268 is methylated, the restriction enzyme HinfI cleaves the PCR product into two fragments of 29bp and 126bp. Non-methylated 25S rRNA has a T at position 2268 after bisulfite treatment, and this eliminates the HinfI restriction enzyme site, leaving the product at 155bp and undigested. Undigested PCR products were used as loading controls. Primer sequences used are provided (Additional file 1: Table S6).

### Semi-quantitative PCR

Semi-quantitative PCR was performed using an Invitrogen SuperScript III kit as per the manufacturer’s recommendations from 2 μg of total RNA and oligo-dT primed cDNA synthesis. Semi-quantitative PCR detection of *TRM4A, TRM4B, NSUN5, NOP2B* and *NOP2C* mRNA was performed using the primers provided (Additional file 1: Table S6). For the RNA input control, amplification of the housekeeping gene PDF2A was used with primers forward PDF2_RT-PCR_F and reverse PDF2_RT-PCR_R. Quantitative PCR was performed to test NOP2C mRNA abundance using Roche LightCycler480 and SYBER green.

### Availability of supporting data

The data sets supporting the results of this article are available in NCBI’s GEO database repository, and are accessible through GEO Series accession numbers GSE68444, GSE68445, GSE68447 and GSE68448.

## Additional file

Additional file 1:
**Figure S1**. Efficient bisulfite conversion of non-methylated cytosine residues. **Figure S2**. Characterization of *Arabidopsis thaliana* T-DNA mutants. **Figure S3**. Specificity of tRNA and rRNA MTases. **Figure S4**. Multiple sequence alignment of methyltransferase motifs from *Arabidopsis thaliana* RMTases. The amino acid sequences of two subfamilies of RMTases in Arabidopsis; (A) TRM4A and TRM4B; (B) NOP2A/OLI2, NOP2B and NOP2C were aligned using Clustal Omega [72]. **Table S1**. Unique tRNA isodecoder consensus sequences used for tRNA expression and methylation analysis. **Table S2**. Annotation table of tRNA isodecoder sequences detected in diverse plant species. **Table S3**. Ribosomal RNA sequences used for methylation analysis in diverse plant species and number of m^5^C sites. **Table S4**. Methylation % of tRNAs from *A. thaliana*, *B. rapa*, *T. durum*, *C. taxifolia*, *N. occulata* and *G. biloba*. **Table S5**. Methylation % of rRNAs from *A. thaliana*, *B. rapa*, *T. durum*, *C. taxifolia*, *N. occulata* and *G. biloba*. **Table S6**. Primer sequences used in this study [73]. **Table S7**. Read coverage of libraries sequenced. (ZIP 5681 kb)

## Abbreviations

rRNA: Ribosomal RNA
tRNA: Transfer RNA
m^5^C: 5-methylcytosine
RMTase: RNA methyltransferase
TRM4: Transfer RNA methyltransferase 4
TRDMT1: Transfer RNA Asp methyltransferase 1
RNA-seq: Illumina RNA sequencing
RBS-seq: Illumina RNA BiSulfite sequencing
NOP2: Nucleolar Protein 2
OLI2: Oligocellula 2
NSUN2: NOP2/Sun domain protein 2
NSUN5: NOP2/Sun domain protein 5
BS: Bisulfite
R-Luc: Renilla luciferase
RCMT9: RNA cytosine methyltransferase 9
AdoMet: S-adenosyl-L-methionine
dCAPS: Derived cleaved amplified polymorphic sequences
PAR: Photosynthetic active radiation
