# Supplementary material for: Conservation of tRNA and rRNA 5-methylcytosine in the kingdom Plantae
Source: BMC Plant Biol. 2015 Aug 14;15:199. doi: 10.1186/s12870-015-0580-8 (PMC4535395; doi:10.1186/s12870-015-0580-8)
Supplement: Additional file 1: — Figure S1. Efficient bisulfite conversion of non-methylated cytosine residues. Figure S2. Characterization of Arabidopsis thaliana T-DNA mutants. Figure S3. Specificity of tRNA and rRNA MTases. Figure S4. Multiple sequence alignment of methyltransferase motifs from Arabidopsis thaliana RMTases. The amino acid sequences of two subfamilies of RMTases in Arabidopsis; (A) TRM4A and TRM4B; (B) NOP2A/OLI2, NOP2B and NOP2C were aligned using Clustal Omega [72]. Table S1. Unique tRNA isodecoder consensus sequences used for tRNA expression and methylation analysis. Table S2. Annotation table of tRNA isodecoder sequences detected in diverse plant species. Table S3. Ribosomal RNA sequences used for methylation analysis in diverse plant species and number of m5C sites. Table S4. Methylation % of tRNAs from A. thaliana, B. rapa, T. durum, C. taxifolia, N. occulata and G. biloba. Table S5. Methylation % of rRNAs from A. thaliana, B. rapa, T. durum, C. taxifolia, N. occulata and G. biloba. Table S6. Primer sequences used in this study [73]. Table S7. Read coverage of libraries sequenced. (ZIP 5681 kb) [file 12870_2015_580_MOESM1_ESM.zip › Figure_S4.pdf]

A

```
TRM4A_At4g40000  ETEAGNMTRQESVSMIMFISN---------------------------------F
TRM4B_At2g22400  ENEVGNITRQEAVSMVPPLFLDVHPDHFVLDMCAAPGSKTFQLLEIIHEASEPGS
```
Motif X   Motif I

```
TRM4A_At4g40000  VWLNQVVANDVDYKRSNLLIHQTKRTCTTNLMVTNNEGQHFPSCNTKRTLSV-AS
TRM4B_At2g22400  LPNGLVVANDVDFKRSNLLIHQTKRMCTSNLIVTNHEGQQFPGCRLNKSRASEKG
```

```
TRM4A_At4g40000  ETNPHPIDQLLFDRVLCDVPCSGDGTLRKAPDIWRRWNSGSGNGLHSLQVVLAMR
TRM4B_At2g22400  ISENMPINQLAFDRVLCDVPCSGDGTLRKAPDIWRKWNSGMGNGLHSLQIILAMR
```
Motif IV

```
TRM4A_At4g40000  GLSLLKVGGRMVYSTCSMNPIEDEAVVAEILRRCGCSVELVDVSDKLPELIRRPG
TRM4B_At2g22400  GLSLLKVGGKMIYSTCSMNPVEDEAVVAEILRRCGDSVELLDVSDKLPELIRRPG
```
Motif VI

```
TRM4A_At4g40000  LTKWKVHDRGGWYRSYKDVPKSQRDGVLRSMFPSGKSDKDSSGGKNSY-------
TRM4B_At2g22400  LKAWKVRDKGGWFTSYKDVPQNRRGGVLVSMFPSGKYLKDSTETTEKNENGDVNG
```

```
TRM4A_At4g40000  ---------EEMASISSDESAEEVCDLPLELCMRILPHDQNTGGFFIAVLHK
TRM4B_At2g22400  CEDGLKETDISVVDATPEEQAEEVSDLPLERCMRIIPHDQNTGAFFIAVLQK
```
Motif VIII

B

```
OLI2_At5g55920   SIRTNTLKTRRRDLADVLLNRGVNLDPLSKWSKVGLV---------IYDSQVPIG
NOP2B_At4g26600  SIRTNTLKTRRRDLADILLNRGVNLDPLSKWSKVGLI---------VYDSQVPIG
NOP2C_At1g06560  TLQGLPTDPYYRERSGLYIGMGTAM-----LSRAGMFRVPNGIAVDLNHRVFRLP
```
Motif N1

```
OLI2_At5g55920   ATPEYLAGYYMLQGASSFLPVMALAPRENERIVDVAAAPGGKTTYIAALMKNTGL
NOP2B_At4g26600  ATPEYLAGFYMLQSASSFLPVMALAPREKERVVDMAAAPGGKTTYVAALMKNTGI
NOP2C_At1g06560  SLHNILEGEIFLQNLPSIIVAHALDPQKGERILDMCAAPGGKTTAIAILMNDEGE
```
Motif X   Motif I

```
OLI2_At5g55920   IYANEMKVPRLKSLTANLHRMGVTNTIVCNYDGR---------------------
NOP2B_At4g26600  IYANEMKVPRLKSLSANLHRMGVTNTIVCNYDGR---------------------
NOP2C_At1g06560  IVAADRSHNKVLVVQNLSAEMGFTCITTCKLDALKSVCLPTTLNESTILINGDNS
```

```
OLI2_At5g55920   -------------------------------------------------------
NOP2B_At4g26600  -------------------------------------------------------
NOP2C_At1g06560  SSMTSHSELSSNEEMTSVTSRRSEADKSCEKNDSTEQPNGGDNVSQAYIRKNKGR
```

```
OLI2_At5g55920   ---------------ELPKVLGQNTVDRVLLDAPCSGTGIISKDESVKITKTMDE
NOP2B_At4g26600  ---------------E-----------------------VISKDESVKTSKSADD
NOP2C_At1g06560  LKNGRGRTQCQGGRAGKSQGFPPNSFDRVLLDAPCSALGLRPRLFA--GLETVVS
```
Motif IV

```
OLI2_At5g55920   IKKFAHLQKQLLLAAIDMVDANSKTGGYIVYSTCSIMVTENEAVIDYALKKRDV-
NOP2B_At4g26600  IKKFAHLQKQLILGAIDLVDANSKTGGYIVYSTCSVMIPENEAVIDYALKNRDV-
NOP2C_At1g06560  LRNHGWYQRKMLDQAVQ----LVRVGGILVYSTCTINPSENEAVVRYALDKYRFL
```
Motif VI

```
OLI2_At5g55920   KLVTCGLDFGRKGFTRF-------REHRFQPSLDKT-RRFYPH-VHNMDGFFVAK
NOP2B_At4g26600  KLVPCGLDFGRPGF----------REHRFHPSLEKT-RRFYPH-VHNMDGFFVAK
NOP2C_At1g06560  SLAPQHPRIGGPGLVGRCEFPDGYIEEWLKPGEEELVQKFDPSSELDTIGFFIAK
```
Motif VIII

```
OLI2_At5g55920   LKK
NOP2B_At4g26600  LKK
NOP2C_At1g06560  FSV
```
