# Supplementary figures and images for: Conservation of tRNA and rRNA 5-methylcytosine in the kingdom Plantae
Source: BMC Plant Biol. 2015 Aug 14;15:199. doi: 10.1186/s12870-015-0580-8 (PMC4535395; doi:10.1186/s12870-015-0580-8)

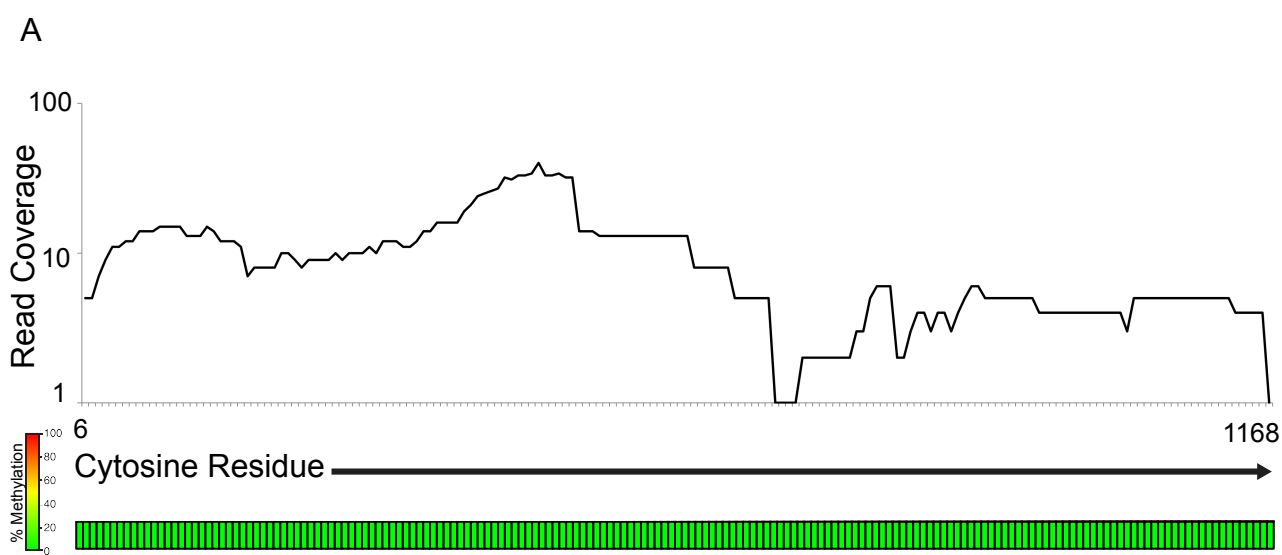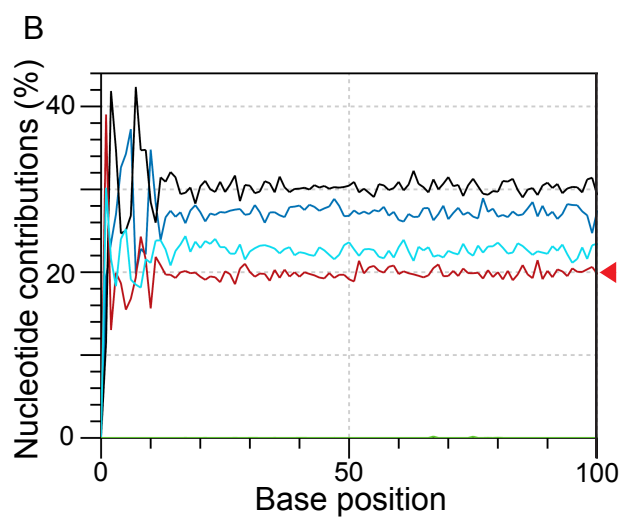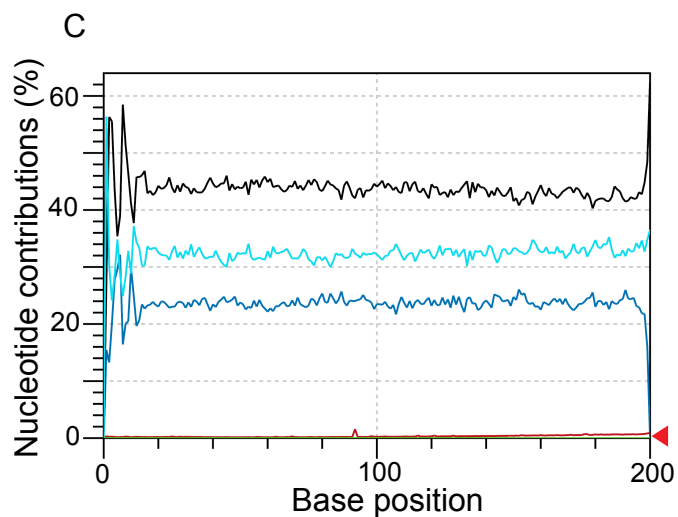

— Ambiguous  
— Guanine  
— Cytosine  
— Thymine  
— Adenine

Supplement: Additional file 1: — Figure S1. Efficient bisulfite conversion of non-methylated cytosine residues. Figure S2. Characterization of Arabidopsis thaliana T-DNA mutants. Figure S3. Specificity of tRNA and rRNA MTases. Figure S4. Multiple sequence alignment of methyltransferase motifs from Arabidopsis thaliana RMTases. The amino acid sequences of two subfamilies of RMTases in Arabidopsis; (A) TRM4A and TRM4B; (B) NOP2A/OLI2, NOP2B and NOP2C were aligned using Clustal Omega [72]. Table S1. Unique tRNA isodecoder consensus sequences used for tRNA expression and methylation analysis. Table S2. Annotation table of tRNA isodecoder sequences detected in diverse plant species. Table S3. Ribosomal RNA sequences used for methylation analysis in diverse plant species and number of m5C sites. Table S4. Methylation % of tRNAs from A. thaliana, B. rapa, T. durum, C. taxifolia, N. occulata and G. biloba. Table S5. Methylation % of rRNAs from A. thaliana, B. rapa, T. durum, C. taxifolia, N. occulata and G. biloba. Table S6. Primer sequences used in this study [73]. Table S7. Read coverage of libraries sequenced. (ZIP 5681 kb) [file 12870_2015_580_MOESM1_ESM.zip › Figure_S1.pdf]

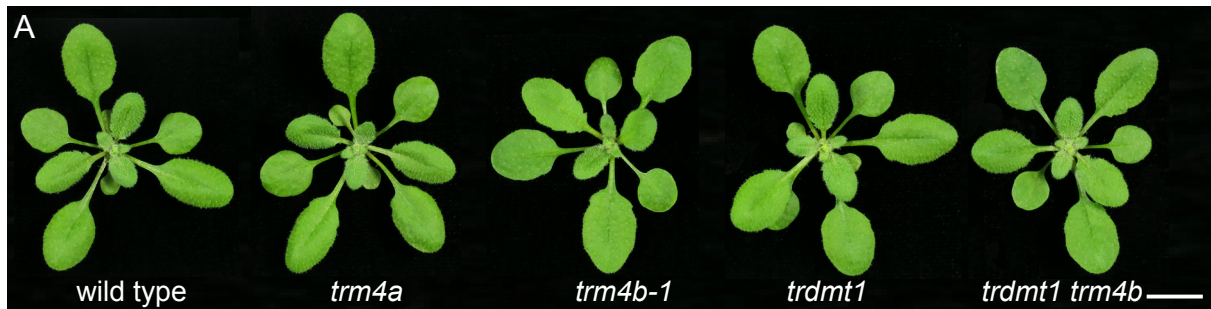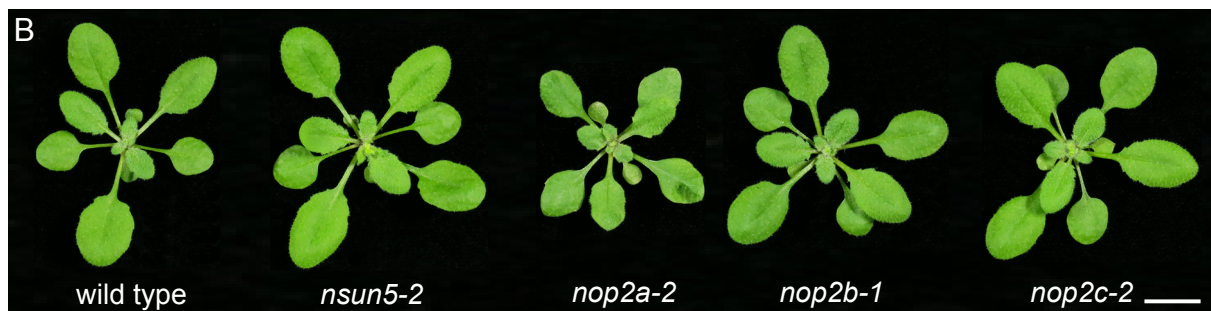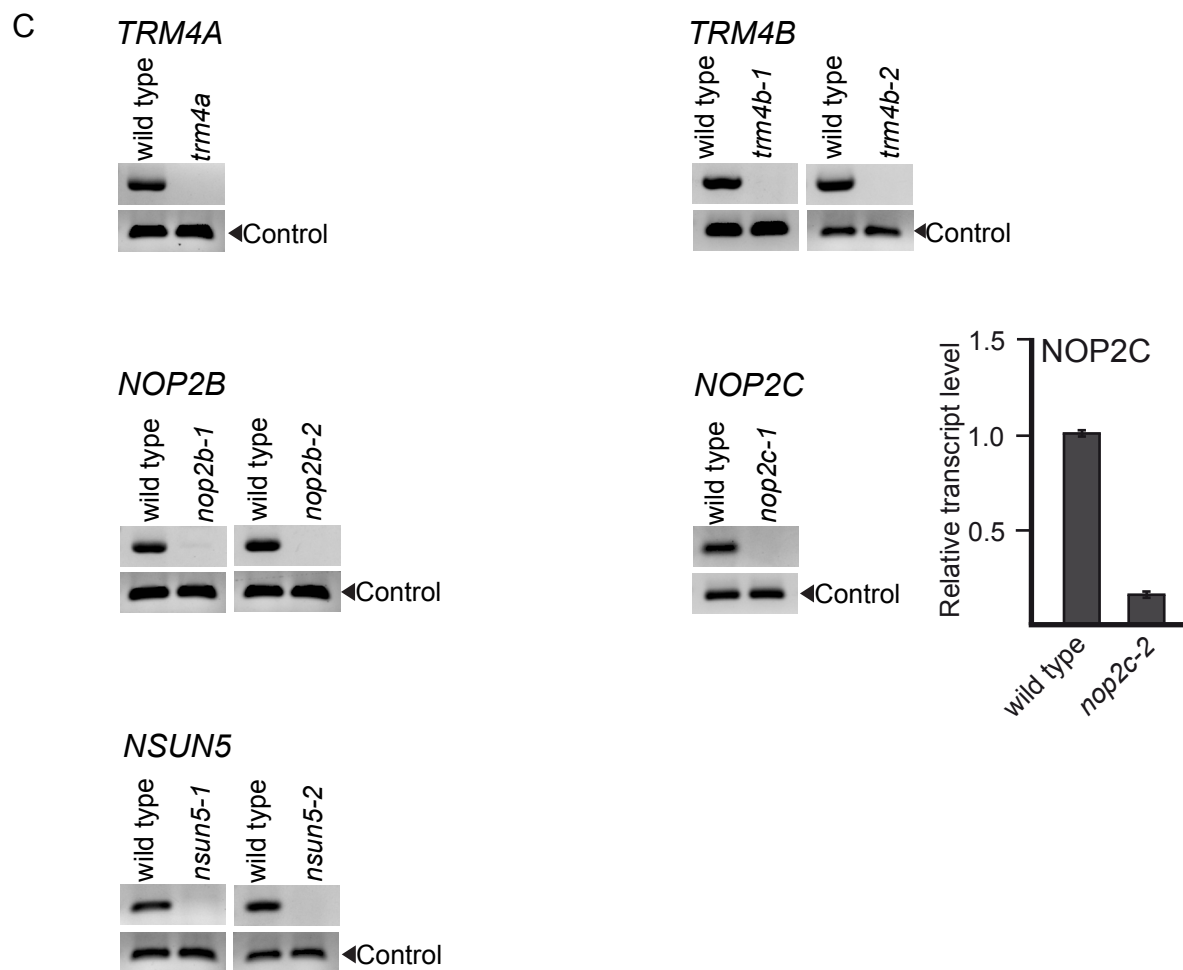

Supplement: Additional file 1: — Figure S1. Efficient bisulfite conversion of non-methylated cytosine residues. Figure S2. Characterization of Arabidopsis thaliana T-DNA mutants. Figure S3. Specificity of tRNA and rRNA MTases. Figure S4. Multiple sequence alignment of methyltransferase motifs from Arabidopsis thaliana RMTases. The amino acid sequences of two subfamilies of RMTases in Arabidopsis; (A) TRM4A and TRM4B; (B) NOP2A/OLI2, NOP2B and NOP2C were aligned using Clustal Omega [72]. Table S1. Unique tRNA isodecoder consensus sequences used for tRNA expression and methylation analysis. Table S2. Annotation table of tRNA isodecoder sequences detected in diverse plant species. Table S3. Ribosomal RNA sequences used for methylation analysis in diverse plant species and number of m5C sites. Table S4. Methylation % of tRNAs from A. thaliana, B. rapa, T. durum, C. taxifolia, N. occulata and G. biloba. Table S5. Methylation % of rRNAs from A. thaliana, B. rapa, T. durum, C. taxifolia, N. occulata and G. biloba. Table S6. Primer sequences used in this study [73]. Table S7. Read coverage of libraries sequenced. (ZIP 5681 kb) [file 12870_2015_580_MOESM1_ESM.zip › Figure_S2.pdf]

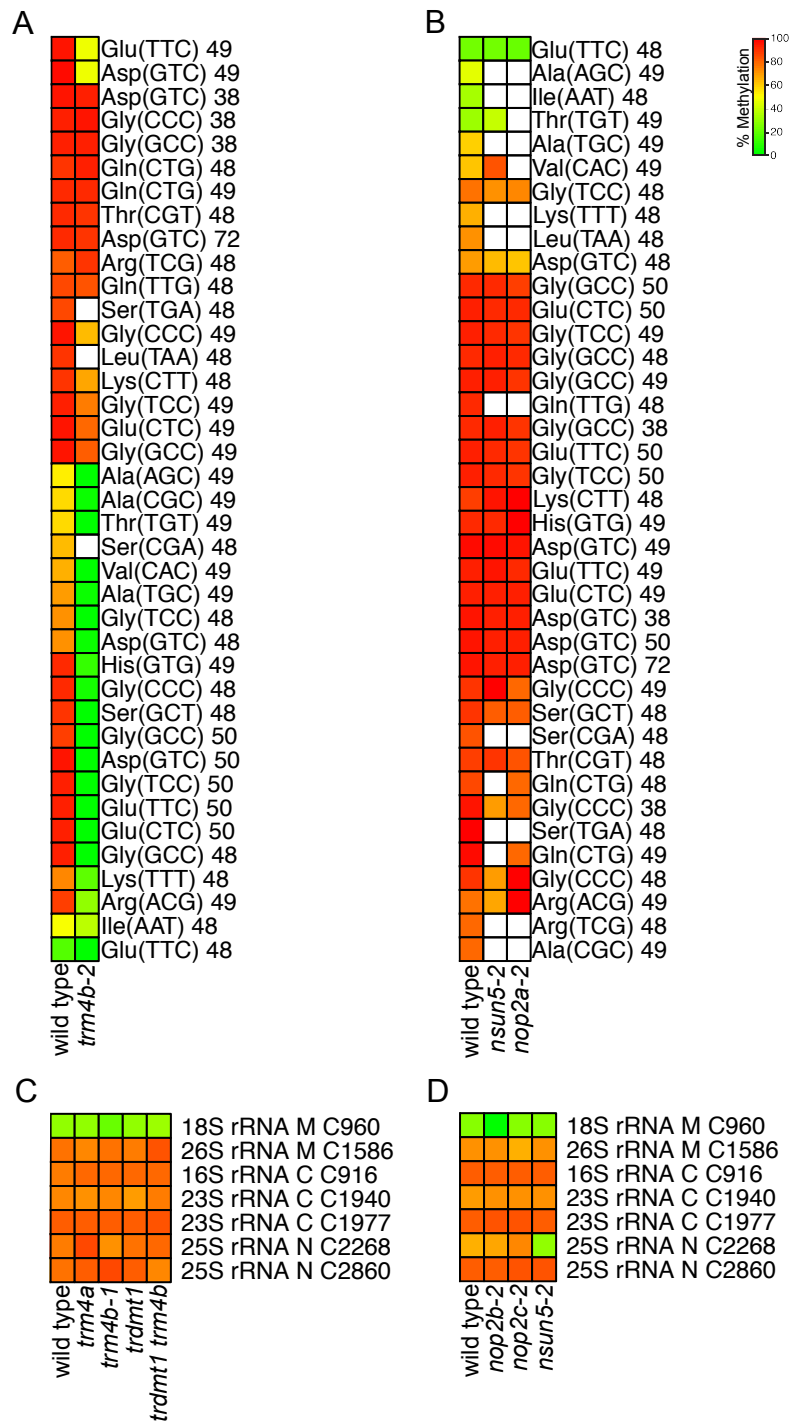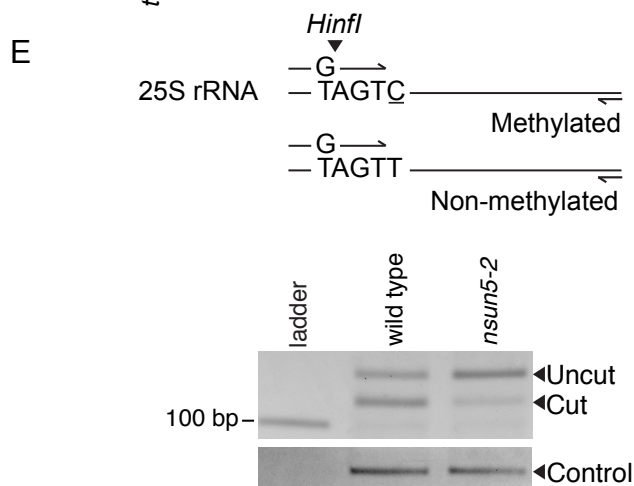

Supplement: Additional file 1: — Figure S1. Efficient bisulfite conversion of non-methylated cytosine residues. Figure S2. Characterization of Arabidopsis thaliana T-DNA mutants. Figure S3. Specificity of tRNA and rRNA MTases. Figure S4. Multiple sequence alignment of methyltransferase motifs from Arabidopsis thaliana RMTases. The amino acid sequences of two subfamilies of RMTases in Arabidopsis; (A) TRM4A and TRM4B; (B) NOP2A/OLI2, NOP2B and NOP2C were aligned using Clustal Omega [72]. Table S1. Unique tRNA isodecoder consensus sequences used for tRNA expression and methylation analysis. Table S2. Annotation table of tRNA isodecoder sequences detected in diverse plant species. Table S3. Ribosomal RNA sequences used for methylation analysis in diverse plant species and number of m5C sites. Table S4. Methylation % of tRNAs from A. thaliana, B. rapa, T. durum, C. taxifolia, N. occulata and G. biloba. Table S5. Methylation % of rRNAs from A. thaliana, B. rapa, T. durum, C. taxifolia, N. occulata and G. biloba. Table S6. Primer sequences used in this study [73]. Table S7. Read coverage of libraries sequenced. (ZIP 5681 kb) [file 12870_2015_580_MOESM1_ESM.zip › Figure_S3.pdf]
